# Comparison of the Accuracy and Clinical Parameters of Patient-Specific and Conventionally Bended Plates for Mandibular Reconstruction

**DOI:** 10.3389/fonc.2021.719028

**Published:** 2021-11-26

**Authors:** Henriette L. Möllmann, Laura Apeltrath, Nadia Karnatz, Max Wilkat, Erik Riedel, Daman Deep Singh, Majeed Rana

**Affiliations:** Department of Oral and Maxillofacial Surgery, University Hospital Duesseldorf, Duesseldorf, Germany

**Keywords:** mandibular reconstruction, CAS, PSI, reconstruction plate, virtual planning

## Abstract

**Objectives:**

This retrospective study compared two mandibular reconstruction procedures—conventional reconstruction plates (CR) and patient-specific implants (PSI)—and evaluated their accuracy of reconstruction and clinical outcome.

**Methods:**

Overall, 94 patients had undergone mandibular reconstruction with CR (*n* = 48) and PSI (*n* = 46). Six detectable and replicable anatomical reference points, identified *via* computer tomography, were used for defining the mandibular dimensions. The accuracy of reconstruction was assessed using pre- and postoperative differences.

**Results:**

In the CR group, the largest difference was at the lateral point of the condyle mandibulae (D2) -1.56 mm (SD = 3.8). In the PSI group, the largest difference between preoperative and postoperative measurement was shown at the processus coronoid (D5) with +1.86 mm (SD = 6.0). Significant differences within the groups in pre- and postoperative measurements were identified at the gonion (D6) [*t*(56) = -2.217; *p* = .031 <.05]. In the CR group, the difference was 1.5 (SD = 3.9) and in the PSI group -1.04 (SD = 4.9). CR did not demonstrate a higher risk of plate fractures and post-operative complications compared to PSI.

**Conclusion:**

For reconstructing mandibular defects, CR and PSI are eligible. In each case, the advantages and disadvantages of these approaches must be assessed. The functional and esthetic outcome of mandibular reconstruction significantly improves with the experience of the surgeon in conducting microvascular grafts and familiarity with computer-assisted surgery. Interoperator variability can be reduced, and training of younger surgeons involved in planning can be reaching better outcomes in the future.

## Introduction

After continuity resections of the lower jaw in case of carcinoma, osteonecrosis, osteomyelitis, or trauma, a mandibular reconstruction is essential to restore function and esthetics (1, 2). The size of the defect is determined by the preoperative extent, the entity of the pathology, and the resulting radicality of the resection.

Defects of the mandible are reconstructable using either a reconstruction plate without bony reconstruction or immediately with a combination of reconstruction plate and primary bone flap. For reconstructing with a fibula flap, hand-bended (conventional reconstruction) plates (CR) or patient-specific implants (PSI) can be used. Despite the considerable progress in microvascular surgery, complications, such as tissue necrosis, failure of the graft, infections (donor site or recipient), prolonged hospital stay, and a long recovery process, occur (3, 4).

Advancements in computer-assisted surgery (CAS), particularly with regard to computer-aided design/computer-aided manufacturing (CAD/CAM) technology, are beneficial compared to the traditional method of mandibular reconstruction with hand-bent plates (5–15). The accuracy of CAD/CAM or selective laser melting plates is superior to the manually bent reconstruction plates. These plates provide greater results in terms of strength and intraoperative positioning (16). The decisive factors for this procedure are anatomical and symmetrical bone shaping, restoration of a stable dental occlusion, and condylar repositioning into a centric relation (5, 17–19).

In the conventional technique, in contrast to CAS, the plates are bent intraoperatively or preoperatively manually before their adaptation. Depending on the complexity of the case and the skills as well as experience of the surgeon, this procedure might be very time-consuming. The standard plates offered by manufacturers do not always possess the required size and number of holes for the intraoperative situation. An advantage is offered by the PSI, which are more resistant to fracture while normally being thinner than CR. PSI do not need to be bent to fit the mandible of the patient and do not require predefined bending points such as with CR (20, 21). With the advancement of CAD/CAM technology, it is possible to accurately plan the reconstruction of craniofacial defects preoperatively, manufacture precise patient-specific implants, and place them in shorter operating times. The implant can be designed and shaped by the surgeon according to the defect size, shape, and morphology (22, 23). By selecting the appropriate design method, manufacturing process, and implant material, it is possible to perform a precise surgical procedure and reduce complications (24–31). The integration of this technology in the pre- and intraoperative workflow has simplified the production of cutting guides and has been shown to shorten the operation time and the length of stay and to improve osseus consolidation, symmetry, and morphology (10, 29, 32). Recent research demonstrated additional advantages, for instance, minimized interoperator variability caused by the experience of the surgeon and improved teaching possibilities for younger colleagues involved in the planning procedures/sessions with a senior consultant and/or biomedical engineer (33).

The preoperative planning of the exact position of dental implants is enabled for an early satisfying functional outcome (22). In contrast to the reported advantages, the time-consuming preoperative planning and associated costs need to be considered. With precise preoperative planning, it is challenging to react to unexpected intraoperative changes. Implementing changes in virtual planning is complicated (33, 34) and might increase the risk of R1 resections in tumor surgery in cases of primary reconstruction. An R1 resection is the macroscopic removal of the tumor. In histopathology, however, smaller portions of the tumor can be detected in the resection margin. The aim of this study was, on the one hand, to compare the results of patients regarding surgical technique and, on the other hand, to evaluate the accuracy and reproducibility of virtual surgical planning.

The use of modern technologies offers novel possibilities in the treatment of complex defect situations. With the development of preoperative virtual planning as well as PSI, another possibility emerged for achieving a true-to-the-original contour of the resected bone. Surgeons and users can use IPS Gate^®^ to request, plan, and complete patient-specific products. The PSI, planning guidance, and anatomical models are manufactured using various materials with the help of the latest construction technologies. Through computer-based planning and functionalized PSI, preoperative planning can be transferred to higher precision of surgeries. For case creation, patient data and case-related information are uploaded to the web-based IPS Gate^®^ platform. Based on the information and requirements of the users, the IPS^®^ developer prepares the case planning in close collaboration with the surgeon. Once the resection margins have been defined, the donor region is virtually projected onto the recipient region, and the graft is designed for the best possible esthetic and functional restoration. Drilling and marking templates as well as a case-specific optimized implant are created. The type, diameter, and length of the osteosynthesis screws are defined. Finally, the surgeon approves the design for production.

In the study at hand, we analyze the restoration of the mandible with a PSI or with the conventional technique regarding their accuracy of reconstruction, the associated complication rate, and the outcome. To evaluate the reconstruction, six clinically relevant distances of the preoperative and postoperative conditions were evaluated. This is a less-followed approach but highly suitable for determining pre- and post-operative dimensions. With our research, clinicians comprehend the extent to which a PSI or conventional reconstruction is advisable for recovery with low complication rates and improved outcomes.

## Materials and Methods

This study was approved by the local ethics committee at the University of Düsseldorf, Germany (approval number 2018-250). In this retrospective study, the results of mandibular reconstructions of osseous defects treated with patient-specific or conventional implants in 2014–2019 in the Department of Oral and Maxillofacial Plastic Surgery at the University Hospital Düsseldorf were evaluated.

### Inclusion Criteria

All patients who underwent mandibular reconstruction at the Center for Operative Medicine II Clinic for Oral and Maxillofacial Plastic Surgery at the University Hospital Düsseldorf between 2014 and 2019 were included. Both alloplastic reconstructions and free microvascular grafts were included in this study. All cases operated on using the standard method with CR (hand-bended reconstruction plates) and those who received a PSI were included, as well as cases from secondary reconstruction.

### Acquisition of Patient Data

Based on patient data, the course, the healing process, and the postoperative quality of life are compared. A group of 46 patients (PSI) were compared to 48 patients who were treated with a conventional procedure (CR). The following patient- and implant-related data were collected from clinical documentation, surgical reports, and findings:

- patient data (name, age, date of birth, and gender)
- preoperative findings (previous operations, concomitant diseases/pretreatments with possible effect on wound healing, etiology of the defect, histology of the defect, preoperative radiological findings, localization of the defect, and size of the defect)
- surgery (date, type of graft resection limits, ischemia time, duration of surgery, surgical technique, implant material, intraoperative fitting accuracy, complications during insertion and fixation of the implant, and necessary adjustment of the bony graft bed or the implant)
- inpatient stay (wound healing process, postoperative imaging, position of the implant, symmetry of the reconstruction, signs of loosening, complications, and length of stay)
- postoperative course (sensitivity disorders, pain, pressure sensitivity, skin conditions, scar conditions, and complications)
- preoperative and postoperative intercondylar distance

### Selection of CT Data Sets

The preoperative CT image should accurately depict the current condition before partial mandibular resection and allow the measurement of the defined measurement distances. The postoperative dataset was the first postoperative image to show all relevant anatomical structures. For evaluation, the distances of the corresponding points were measured pre- and postoperatively and tabulated. In addition, the differences of the distances were determined by subtracting the postoperative value from the value determined preoperatively.

### Determination of the Defect Extent and Size

To localize the osseous defect or lesion, the bony and soft tissue portions of the mandible were divided into sections. In the bony portion, four areas were defined according to the anatomy: the alveolar process (pars alveolaris mandibulae), the ascending branch (ramus mandibulae), the mandibular angle (angulus mandibulae), and the mandibular body (corpus mandibulae). If partial resection of the mandible is indicated, the sections can be resected individually or in combination, as well as unilaterally or bilaterally. The soft tissue portion of the mandible was divided into the tongue, floor of the mouth, cheek, and lip for localization in the presence of a lesion. With the aid of histopathology, radiology, or surgical reports, the defect size and localization were documented.

### Measuring Points and Distances

**Figure 1:**
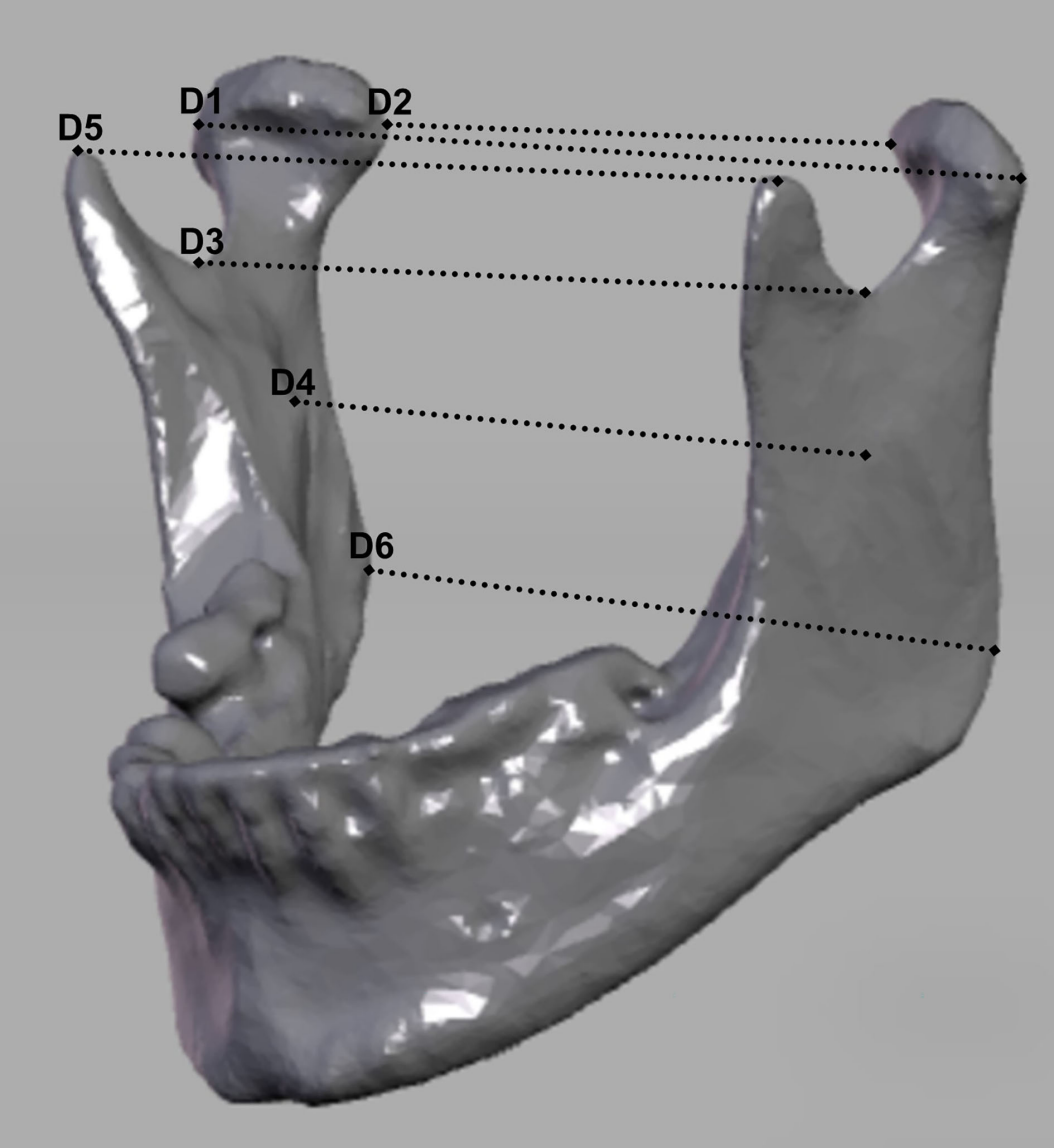
Representation of the measurement distances. D1, lateralmost point of condyle mandibulae right to the lateralmost point of condyle mandibulae left; D2, medialmost point of condyle mandibulae right to the medialmost point of condyle mandibulae left; D3, most caudal point of the incisura mandibulae on the right to the most caudal point of the incisura mandibulae on the left; D4, foramen mandibulae on the right to the foramen mandibulae on the left, D5, processus coronoideus on the right to processus coronoideus on the left; D6, gonion on the right to gonion on the left.

To evaluate the reconstruction, six clinically relevant distances of the preoperative and postoperative situation were compared. The dataset was oriented according to the Frankfurt horizontal plane and mid-sagittal plane. Measurements were conducted from the capitulum (most lateral and most medial points), incisura (most caudal points), mandibular foramina, to the coronoid process (most cranial points), dorsal tip of the mandible closest to the gonion point) using preoperative and postoperative CT or CBCT. They run between the following bilateral anatomical structures: D1, capitulum mandibulae lateralmost point; D2, capitulum mandibulae medialmost point; D3, incisura mandibulae; D4, foramen mandibulae; D5, processus coronoideus; and D6, gonion dorsalmost tip of the mandible (cf. **Figure 1**). The measurements were captured using dicomPACS^®^.

To evaluate the accuracy of the reconstruction, the distances measured pre- and postoperatively were compared. This enables verifying whether the original position of the condyles and the symmetry of the mandible have been restored for ensuring functionality and esthetics. The restoration of the individual dimensions is a key quality factor after resection and the reconstruction of the mandible. Whether a narrowing or widening of the respective measured distance between the corresponding anatomical points has occurred postoperatively is reflected in the sign of the calculated difference. A deviation into the negative range means a narrowing of the section in question, while a deviation into the positive range is to be interpreted as a widening in this area. If the differences calculated for a patient have positive and negative signs, this means that opposing movements have taken place during the reconstruction, *i*.*e*., the mandibular segments have rotated.

### Statistical Analysis

The determined values of the measurements as well as the clinical data were statistically analyzed using jamovi (version 1.6.9). The Shapiro–Wilk test was conducted to check the data for normal distribution, and the Mann–Whitney *U*-test and Student’s *t*-test were used to compare the means of the two groups. The Mann–Whitney *U*-test is a parameter-free statistical test that compares two independent samples that are not normally distributed. Thus, it is used to test the significance of the consistency between two distributions. A *p*-value of <.05 was defined as significant, a value of <.01 as very significant, and a value of <.001 as highly significant. A significance level of *p* >.05 is set for hypothesis testing. For associations between two variables, like A and B, Pearson’s product–moment correlation (*r*) is calculated if the assumptions of linear relationship and exclusion of outliers tested by visual inspection of scatterplots and normal distribution of data, assessed with Shapiro-wilk test, were met. For non-normal data, Spearman’s rank-order correlation (*ρ*) is calculated. Mean differences are tested with independent *t*-test when significant outliers, identified with boxplots, were excluded and normal distribution of the dependent variable, tested with Shapiro–Wilk test, and homoscedasticity, tested with Levene’s test, were met. Mean differences of non-normal dependent variable data were analyzed with Mann–Whitney *U*-test.

## Results

### Descriptive Statistics

The patient collective was distributed in two groups and analyzed according to age at the time of surgery, gender, clinical picture, and resection size. The collective consists of 94 patients. Forty-six patients received a PSI, and forty-eight patients were treated with a CR. (cf. **Table 1**).

**Table 1:** Descriptive statistics [conventional reconstruction plates (CR) *vs*. patient-specific implants (PSI)].

	CR	PSI	Total
Patients			
*N*	48	46	94
Age			
Years (mean ± SD)	66.5 ± 13.6	66.2 ± 11.3	66.3 ± 12.5
>60 years	64.6%	67.7%	65.9%
<60 years	35.4%	32.3%	34.1%
Gender			
Male	26 (54.2%)	28 (60.8%)	54 (57.4%)
Female	22 (45.8%)	18 (39.1%)	40 (42.6%)
Diagnosis			
SCC	25	20	45 (47.8%)
Maxillary necrosis	10	8	18 (16.1%)
Pathological fracture	10	6	16 (17.0%)
Fracture	0	2	2 (2.0%)
Osteomyelitis	0	3	3 (3.1%)
Basal cell carcinoma	0	1	1 (1.06%)
Secondary reconstruction	2	6	8 (8.0%)
Complications			
Mean ± SD	1.5 ± 1.7	2 ± 1.9	1.78 ± 1.8
*N* with complications	31 (48.0%)	33 (51.0%)	64 (68.1%)
Defect size (mm)			
Mean ± SD	58.8 ± 28.1	64.7 ± 29.2	61.7 ± 28.7
Maximum	120	120	120
Minimum	8	12	8
Operative time (min)			
Mean ± SD	397 ± 229	467 ± 240	431 ± 236
Maximum	1000	878	1000
Minimum	143	73	73
Hospital stay (days)			
Mean ± SD	26.4 ± 22.4	34.6 ± 32.4	
Maximum	97	125	
Minimum	4	6	
No reconstruction			
Mean ± SD	10.7 ± 8.4	27.1 ± 43.4	16.4 ± 26.5
Local reconstruction			
Mean ± SD	16.3 ± 8.8	27.1	15 ± 8.8
Microvascular reconstruction			
Mean ± SD	35.4 ± 24	36.7 ± 30.6	36.1 ± 27.8
Reconstruction group			
No reconstruction			20 (21.3%)
*N* complications (mean ± SD)			0.9 ± 1.39
Local reconstruction			7 (7.4%)
*N* complications (mean ± SD)			1.29 ± 1.8
Microvascular reconstruction			67 (71.3%)
*N* complications (mean ± SD)			2.07 ± 1.81
Tumor patients			
*N* (% of total)	25 (26.3%)	18 (19.1%)	43 (45.7%)
*N* complications (mean ± SD)	2.2 ± 1.8	2.56 ± 2.04	Tumor: 2.35 ± 1.9
			No tumor: 1.3 ± 1.5

The age of the patients was between 37 and 90 years (MD* = *66.3; SD* = *12.5) and distributed as normal. In the PSI group, the age ranged between 42 and 82 years (MD* = *66.2; SD* = *11.3), with 28 (60.9%) being male. Within the group of patients receiving the CR, the patients were between 37 and 90 years old (MD *= *66.5; SD* = *13.6), of which 54 (57.4%) were male. The mean age of groups PSI and CR did not differ significantly.

All patients were divided according to medical conditions that led to the indications of partial mandibular resection and subsequent reconstruction. Forty-five subjects were diagnosed with squamous cell carcinoma (SCC) (47.8%), and 20 received reconstruction with a PSI (44.4%). In each case, eight of 18 patients (44.4%) followed a diagnosis of maxillary necrosis with reconstruction using a PSI. A pathologic fracture was present in 17.0% (*n = *16) of cases. Fracture was diagnosed in 2.0%, (*n = *2), secondary reconstruction in 9.0% (*n = *9), and osteomyelitis in 3.1% (*n = *3) of patients. One patient developed basal cell carcinoma. In the patient population treated with PSI, four diagnoses occurred: SCC, osteomyelitis, secondary reconstruction, and pathological fracture.

Of the total of 46 patients who received a PSI, 20 (43.5%) had a resection indication based on the diagnosis of squamous cell carcinoma. In contrast, 25 patients with the same diagnosis were treated conventionally. Of the total of 18 patients diagnosed with mandibular necrosis, eight (44.4%) were treated with PSI, and 10 (55.6%) patients received a conventional reconstruction plate.

To investigate associations between a previous disease and a specific diagnosis, a differentiation was made between seven relevant previous diseases. These included cardiovascular, pulmonary, and metabolic diseases, nicotine and alcohol abuse, mental and neurological diseases, and the occurrence of another carcinoma. Considering the presence of previous diseases in each diagnostic group, it was found that 41 of 45 (91.1%) patients who developed squamous cell carcinoma had one or more previous diseases. All patients diagnosed with osteonecrosis of the jaw (*n = *18) had a preexisting disease prior to this diagnosis. Patients with osteomyelitis (*n = *3) or basal cell carcinoma (*n = *1) had one or more prior diseases. In 33.3% (*n = *3) of the cases, no previous disease was present in the secondary reconstructions. Using *χ* ^2^ test, no stochastic dependence between the general presence of a previous disease and a specific diagnosis was demonstrated [*χ* ^2^(1,94)* = *10.9; *p = .*09].

Fourteen of 18 patients diagnosed with osteonecrosis of the jaw, another carcinoma, were already present in terms of medical history, and a stochastic dependency between a prior disease and diagnosis was found [*χ* ^2^(1;94)* = *11.3; *p *= .002]. There was a stochastic dependence likewise between the diagnosis of pathologic fracture and the prior disease of nicotine abuse [*χ* ^2^(1;94)* = *6.2; *p *= .013]. In the sample, an average of *M* = 1.78 complications occurred (SD* = *1.8).

On average, patients who received a PSI had two complications in the postoperative course (SD* = *1.9). Patients who received a CR developed an average of 1.5 complications (SD* = *1.7). The maximum number of complications was six in the PSI patient group and five in the CR patient group. No significant mean difference was tested with an achieved power of 15.8%.

The documented and possible complications that may occur after reconstruction include dehiscence, the development of hematoma, exposed reconstruction plate, dysphagia, necrosis, infection, flap congestion, chyle fistula, restricted mouth opening, recurrence, revision, patient death, dislocation of the reconstruction plate, fistulation, and wound healing problems at the graft harvest site. The mean number of complications in the PSI and CR groups was not significantly different (*U = .*57; *p* >.05).

Overall, postoperative complications occurred in 64 of 94 patients (68.1%), and no complications occurred during follow-up in 30 patients (31.0%) after the use of PSI or CR. Of the patients with documented complications during the postoperative course, 48.4% (*n = *31) had received a CR and 51.6% (*n = *33) a PSI. No stochastic dependence could be determined between the occurrence of complications and the use of a PSI or CR. The probability of revision is not statistically dependent on diagnosis in the sample.

### Mortality

Of the 45 patients diagnosed with SCC, 22.2% (*n = *10) died. A stochastic dependence between the diagnosis of squamous cell carcinoma and the complication death could not be demonstrated using the chi-square test [*χ* ^2^(1;94)* = *3.7; *p *= .056]. The complication death was also not dependent on any of the diagnoses. Statistically, in patients in whom the complication of revision was documented, reconstruction ended lethally in the further course [*χ* ^2^(1;94)* = *5.6; *p *= .018].

### Defect Size

**Figure 2:**
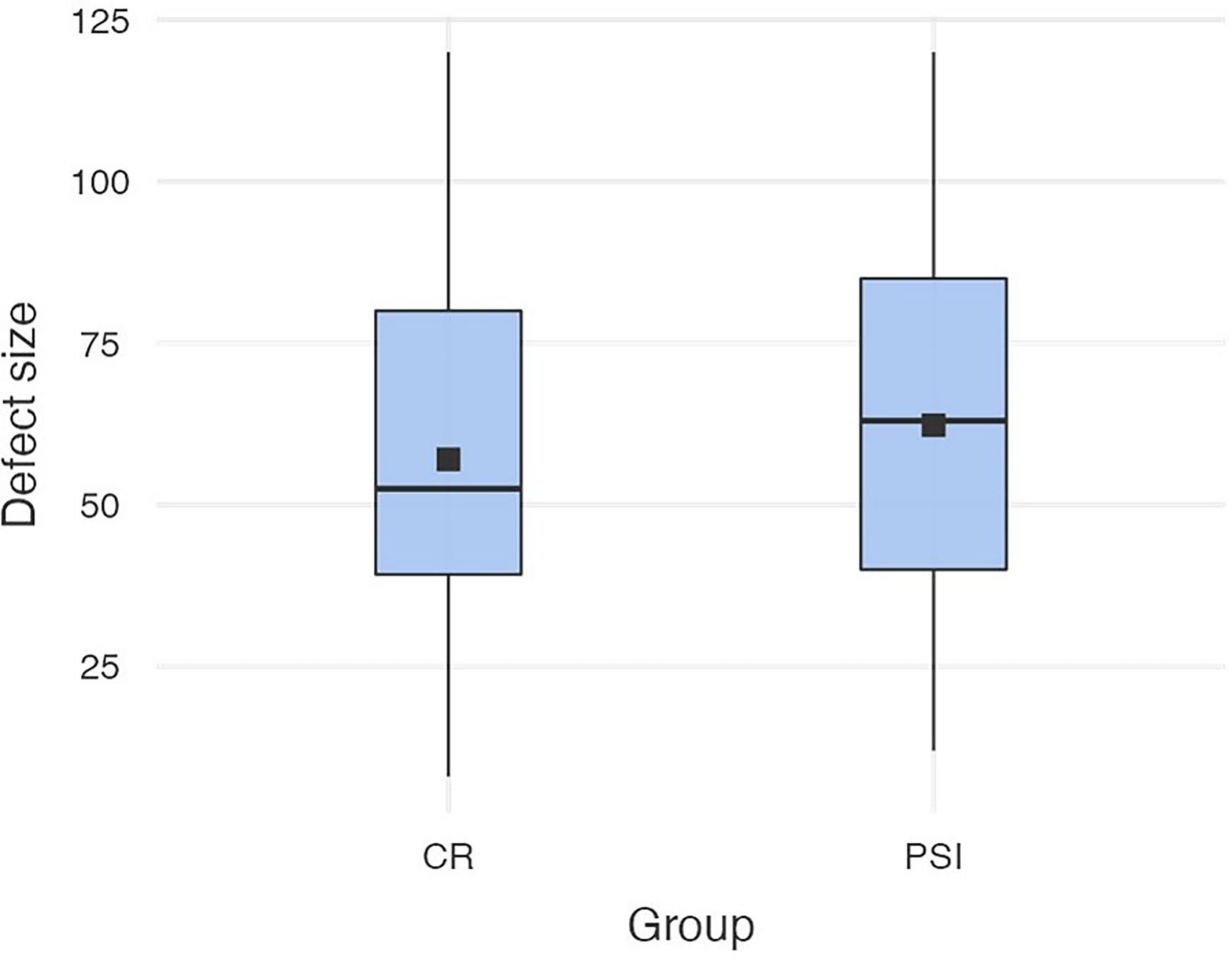
Defect sizes in both groups (conventional reconstruction plates *vs*. patient-specific implants).

The defect size of the collective varies from the minimum extension of 8 mm to a maximum of 120 mm (M *= *61.7; SD* = *28.7). In the patient-specific implant group, the size varies from the minimum extension of 12 mm to a maximum size of 120 mm (M *= *64.7; SD* = *29.2), which was not significantly different to the defect size of the conventional reconstruction plate group that varies from 8 to 120 mm (M* = *58.8, SD* = *28.1) (cf. **Figure 2**).

### Operating Time

**Figure 3:**
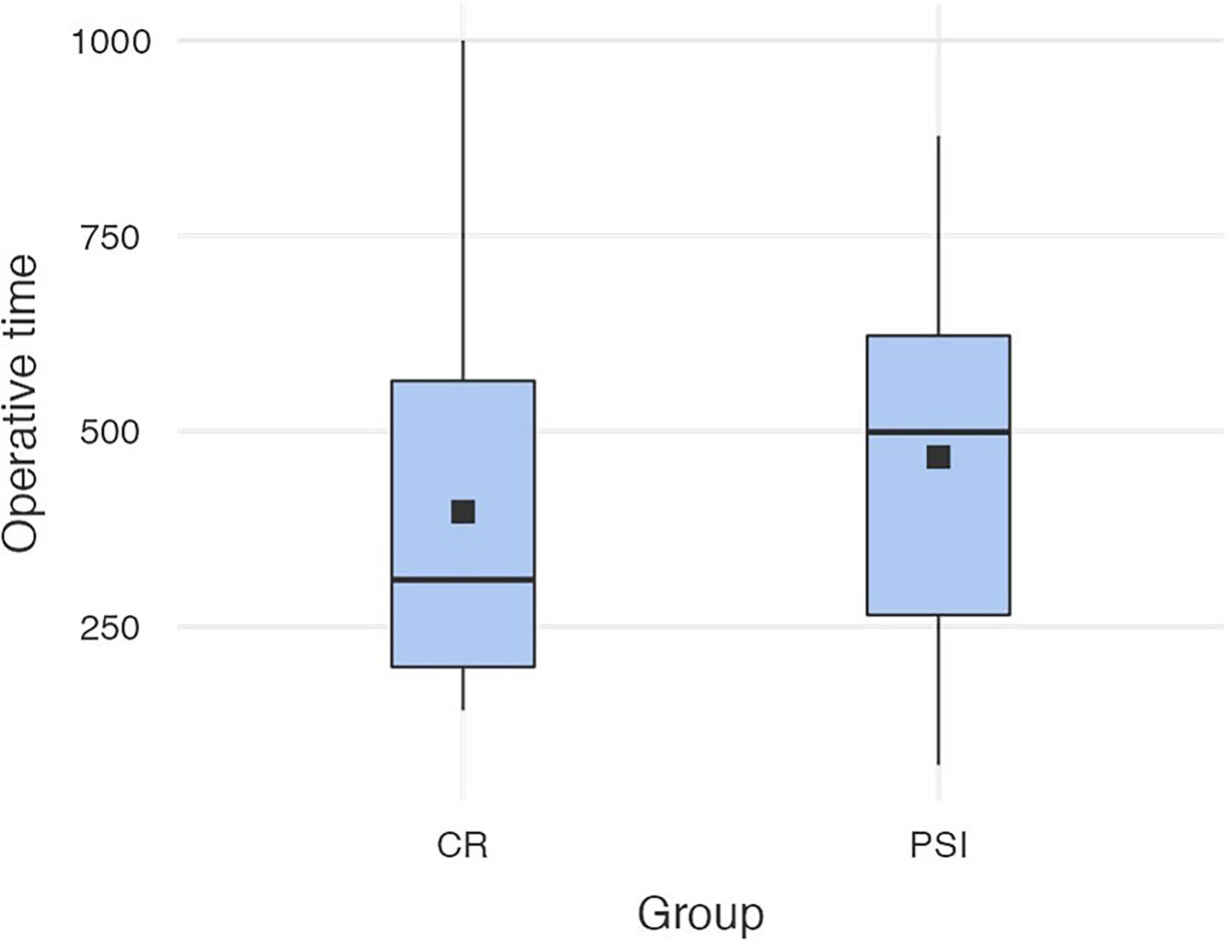
Operation times in both groups (conventional reconstruction plates *vs*. patient-specific implants).

The mean operating time (OR) time of the sample was 431 min (SD *= *236 min), the maximum OR time was 1,000 min, and the shortest OR time was 73 min. The mean OR time of PSI (M = 467 min, SD = 240 min) did not differ significantly to the OR time of CR (M = 397 min, SD = 229 min) [*t* (89)* = *-1.42; *p = .*159) (cf. **Figure 3**).

### Length of Stay

The average length of stay of the group with PSI was 34.6 days (SD* = *32.4) and was not significantly different to the group with CR which was 26.4 days (SD* = *22.4; *U = *-1.4; *p = .*154). The shortest length of stay for the group with PSI was 6 days, and the longest length of stay was 125 days. For the patients with CR, the shortest length of stay was 4 days, and the longest was 97 days.

The mean values of the lengths of stay of the groups PSI and CR are not significantly different. Comparing the length of stay, the mean length of stay of patients with a microvascular graft is highest at 36.1 days (SD* = *27.8). Patients who did not receive reconstruction stayed on the ward for an average of 16.4 days (SD* = *26.5) and those with local reconstruction for 15 days (SD* = *8.8).

In the group of PSI patients who received a microvascular graft, the mean inpatient length of stay was 36.7 days (SD* = *30.6) and in CR patients 35.4 days (SD* = *24.0). Thus, length of stay for patients in the microvascular reconstruction group did not differ between those who received a patient-specific implant and those who received a conventional reconstruction plate.  

### Reconstruction Groups

Three reconstruction groups and seven reconstruction types are differentiated. The three groups consist of patients who received no reconstruction, local reconstruction, and microvascular reconstruction. Reconstruction options include grafts in the form of lingual, radial, pectoralis, and latissimus dorsi flaps as well as fibula grafts. In addition, combinations of grafts are possible for more complex defects.

In the sample, 21.3% (*n = *20) of patients did not receive any reconstruction, 7.4% (*n = *7) took place locally, and most reconstructions were microvascular at 71.3% (*n = *67). On average, patients who received a microvascular graft developed the most complications (M* = *2.1; SD* = *1.8), followed by those who received local reconstruction (M* = *1.3; SD* = *1.8). Patients who did not receive reconstruction developed the least number of complications on average 0.9 (SD* = *1.4).

Of the 45 patients diagnosed with SCC, 88.9% (*n = *40) received a microvascular graft, two received no reconstruction, and three received local reconstruction, with a dependence between diagnosis and the use of a microvascular graft. In patients with the diagnosis of osteonecrosis of the jaw (*n = *18), in 80% of these cases (*n = *8), a microvascular graft was used. When considering complications in the postoperative course, a stochastic dependence was determined between the use of a microvascular graft and the development of dehiscence as a postoperative complication [*χ* ^2^(1;94)* = *6.3; *p *= .012]. Dehiscence was documented in 31 of the 67 patients who received a microvascular graft. Examination of the dependence between preexisting disease and reconstruction type revealed a stochastic dependence between preexisting disease in the form of alcohol abuse and the use of a microvascular graft [*χ* ^2^(1;94)* = *4.8; *p *= .029]. There were 15 patients diagnosed with the preexisting condition of alcohol abuse who received a microvascular graft. In the local reconstruction group, most patients had a prior cardiovascular disease (*n = *5), four patients had a history of another CA, and three each were affected by nicotine abuse and metabolic disease. The most common prior disease in patients with microvascular reconstruction was also cardiovascular (*n = *33), followed by nicotine abuse (*n = *26), another carcinoma (*n = *25), mental/neurological disease (*n = *18), and alcohol abuse (*n = *15).

Of the 94 patients included in the study, 45.7% (*n = *43) were patients with a tumor disease. Of these 43 patients, 18 received a PSI, and 25 patients received a conventional reconstruction plate. No stochastic dependence was demonstrated between a tumor disease and the use of a PSI [*χ* ^2^(1;94)* = *1.6; *p *= .208].

### Squamous Cell Carcinoma

The number of complications between the group of tumor patients (M = 2.2, SD = 1.8) and the group with no tumor diagnosed (M = 1.4, SD = 1.6) does not differ significantly (*U* = 855, *p* = .051, *r* = .255). Thus, on average, significantly more complications develop in patients with a tumor disease. The mean number of complications in group PSI (M = 2.6; SD* = *2.0) and in group CR (M* *= 2.2; SD* = *1.8) did not differ significantly.

### Differences Between the Measurements for PSI and Conventional Restorations

**Figure 4:**
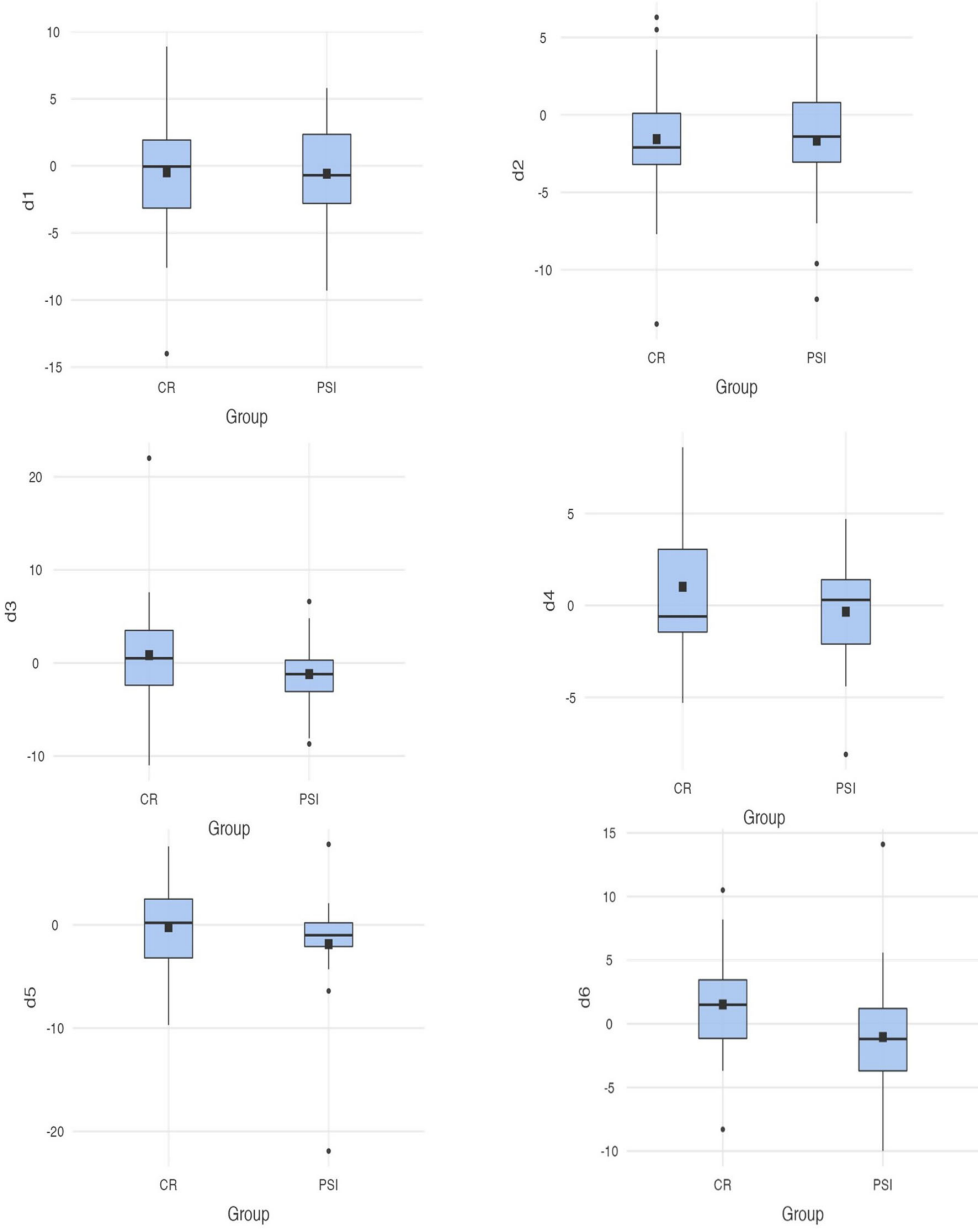
Differences between the measurements (D1–D6) for conventional reconstruction plates and patient-specific implants; D6 [*t*(56)* = *-2.217; *p = .*031 <. 05].

The defined measurement distances run between the following bilateral anatomical structures: D1, capitulum mandibulae lateralmost point; D2, capitulum mandibulae medialmost point; D3, incisura mandibulae; D4, foramen mandibulae; D5, processus coronoideus; and D6, gonion dorsalmost tip of mandible. Post-operative CT scans were, on average, taken 69.6 days after the surgery. Considering the differences of the measurement distances of the group supplied with PSI, on average, the smallest deviations occurred at the measurement point D4 (M* = *-.341; SD *= *3.2) and the largest differences, on average, occurred at the measurement point D5 (*M = *1.9; *SD = *6.0). On average, the measurement points at the coronoid process seem to undergo the greatest change in position during repositioning of the resection parts. In group CR, the largest dimensional changes are drawn at D2 (M =* *-1.6; SD =* *3.8) and the smallest postoperative changes at D5 (M *=* -.217; SD* = *4.2). A direct comparison of the differences in the respective measured distances reveals the following: at D1, an average change of -.48 (SD* = *4.4) is shown in group CR and -.585 (SD* = *4.2) in group PSI. D2 showed an average change of -1.56 (SD* = *3.6) for CR and -1.66 (SD* = *4.1) for PSI, and D3 showed an average change of.838 (SD* = *5.9) for group CR and -1.2 (SD* = *3.8) for group PSI. In D5, group PSI experienced a mean postoperative difference of -1.86 (SD* = *6.0) and group CR -.22 (SD* = *4.2). The mean differences of the differences are significantly different at D6 [*t*(56)* = *-2.217; *p = .*031, *d = .*0286]. In all other measurement stretches, the mean postoperative differences are not significantly different between groups. Point D6 shows the mean postoperative differences of -1.04 (SD* = *4.9) in group PSI and differences of 1.52 (SD* = *3.9), on average, in group CR. Thus, there are significantly lower average postoperative differences between the measurement points at the gonion (cf. **Figure 4**).

### Localization

For a more precise localization of the defect, nine sections were defined. These sections are divided into the tongue, floor of the mouth, alveolar process, mandibular ramus, mandibular angle, mandibular corpus, cheek, lip, and combinations of these localizations. The most frequent complications occurred in patients whose defect was localized to the mandibular corpus. A dependency between defects in the mandibular angle and the postoperative complication of wound healing disorders at the extraction site was found [*χ* ^2^(1;94)* = *11.90; *p *= .049] Another dependency exists between the complication in the form of exposed plates and combined defects [*χ* ^2^(1;94)* = *4.39; *p *= .036]. Complications most frequently occur in patients with defects localized to the floor of the mouth.

### Defect Size Quartile

To validly compare defect sizes and examine them with respect to complications and dependencies on diagnosis, four quartiles were defined. Quartile 1 includes all patients whose defect is in the range of 0–30 mm, quartile 2 includes defects from 31 to 60 mm, all defects from 61 to 90 mm are in the third quartile, and all defects from 91 to 120 mm are in the fourth quartile. A statistical dependence between defects located in the first quartile and the diagnosis of pathologic fracture was determined [*χ* ^2^(1;94)* = *8.87; *p *= .003]. Patients diagnosed with a pathologic fracture statistically had post-resection defects ranging in size from 0 to 30 mm. Similarly, patients had defect sizes in the range of 31-60 mm in 24% of cases because of a diagnosis of squamous cell carcinoma and subsequent resection. A statistical dependence between diagnosis and defect size was demonstrated again [*χ* ^2^(1;94)* = *3.77; *p *= .046]. Regarding the complications occurring post-resection, statistical dependencies between resection size and a complication exist in the first three quartiles. Patients with defect sizes in the range of up to 30 mm developed wound healing disorders at the donor site more frequently in the postoperative course [*χ* ^2^(1;94)* = *8.57; *p *= .003].

## Discussion

Clinical parameters were retrospectively evaluated for obtaining differences in CR and PSI. The virtual planning is intended to support the surgeon in adhering resection limits. The use of computer-assisted planning and manufacturing of PSI should prevent oversizing the extent of resection while achieving R0 status. We investigated how the success of the operation is influenced by pre-existing diseases, previous operations, the size and etiology of the defect, localization, and occurring complications. The question arises as to whether the extensive preoperative virtual planning of the resection procedure leads to certain benefits: a reduced operation time, an optimal fit of the implants without dimensional changes of the bony mandible, and a reduction of the postoperative complication rates for patients.

The results further identified that there is no preferred approach when the defect exceeds a certain size. In the CR group, the mean defect size was 58.8 mm (SD = 28.1) and in the PSI group 64.7 mm (SD = 29.2). There was no significant difference regarding the mean defect size. The decision for or against a PSI is thus not linked to the size of the defect but rather depends on the discretion of the surgeon and the overall complexity of each case in this study. PSI are used primarily for extensive reconstruction (*i*.*e*., multi-fragmentary fibula graft), multi-fragmentary fractures, or pseudarthrosis (35–38). Virtual preoperative planning might simplify the selection of a flap suitable for covering a defect as well as provide the basis for the design, shape, and positioning of the graft based on the previously produced three-dimensional model (39).

In CAD/CAM implants, intraoperative steps can be performed more effectively and efficiently compared to conventional methods in terms of minimizing the burden for patients as well as the risks of postoperative complications (1, 14, 15, 39–41). This approach was also followed by Rustemeyer et al. in their investigation of intraoperative times in osseous reconstructions with free fibular grafts. No significant differences were found between the CAD/CAM group and the conventional implant group (42). This was also shown by Ritschl et al. (43) who also revealed no significant differences between these groups (43). In contrast, other researchers reported shorter operation times in patients with CAD/CAM implants compared to conventionally performed operations (14, 15, 40). The results of this study are consistent with the current literature by Rustemeyer and Ritschl who determined no significant differences in operating times. On average, operations lasted 467 min (SD = 240 min) for patients who received PSI compared to 397 min (SD = 229 min) in the CR group. An exact prefabricated fit for reducing the insertion time of the implant as well as the overall duration of the surgery was not given. However, in case of a more complex operation with a pronounced defect and complex reconstruction, the step of dimensioning the resection margins and the graft can take place preoperatively. Intraoperatively, the prefabricated template makes these steps considerably easier for the surgeon and leads to shorter operation times in the long term (1, 44). In future studies, operations of equivalent complexity should be compared in terms of required time for the individual steps between the two groups—for example, tumor resections in which a radical neck dissection is also performed increase the time required compared to less complex resections. According to a recent study by Vaira et al., unilateral neck dissections require an average operative time of 71.2 min (SD = 27.2 min) (45). Accordingly, the time required doubles for bilateral operations, although variability can be assumed depending on the surgeon. Another component worth to be considered are consolidated workflows through the repeated use of PSI between 2014 and 2020. The growing experience with computer-planned and manufactured implants leads to minimized time for the individual steps. A comparable hypothesis was pursued in a study by Cho et al. who investigated the use of CAD/CAM-assisted surgery for craniosynostosis and concluded that even less experienced surgeons achieved equivalent long-term results with this method (46). Accordingly, gaining knowledge when performing computer-assisted surgery could be proportional to experience.

Comparing the inpatient length of stay of the two groups showed no significant differences. The same conclusion was reached by other researchers who examined the hospital stay between PSI and CR (12, 15, 43). The average length of stay of patients with PSI was approximately 9 days longer compared to patients with CR. Including all patients without differentiation might not yield significant results since, depending on whether reconstruction or resection was performed, the patients require longer recovery under inpatient monitoring. Therefore, a distinction was needed between patients who received a local reconstruction, microvascular reconstruction, and no reconstruction. The group of microvascular grafts showed the longest mean length of stay, followed by the local grafts and the group without reconstruction. Due to the higher complexity of the microvascular reconstructions, this group was divided into PSI and CR patients to compare the healing process and duration based on the inpatient length of stay. However, there were no significant differences between the two groups; this has also been shown by other researchers (13). The healing process might thus be independent of the type of reconstruction used.

The three reconstruction groups were examined regarding dependencies on different diagnoses. A total of 88.9% of patients diagnosed with squamous cell carcinoma received a microvascular reconstruction. An equally high proportion of microvascular grafts (80.0%) is found in patients who underwent resection due to osteonecrosis of the jaw. There is a statistical dependence on the use of a microvascular graft for both the diagnosis of SCC and the diagnosis of osteonecrosis of the jaw (*p* <.05). During the postoperative course, 46.3% of patients who received a microvascular graft developed dehiscence. Considering whether any of the documented pre-existing conditions predisposed to a particular graft, it was found that patients received a microvascular graft after C2 abuses in most cases (*n* = 15). Cardiovascular pathologies were recorded as the most frequent previous disease in the microvascular group. However, this association can also be attributed to the increasing morbidity in older age which also results in a higher susceptibility to developing cardiovascular diseases (47–49).

A total of 45.7% of all patients had a tumor disease, and 41.8% of these tumor patients received a PSI. Especially for patients with complex tumors in the jaw region, preoperative virtual planning can be very important for restoring function and contour (40). Thus, with the help of stereolithic models, the resection margins are precisely dimensioned so that these are tumor-free and less bone is resected (40). Oversizing and resection of healthy bone should be avoided by the CAD/CAM method. Moreover, the implant does not have to be bent to the osseous conditions intraoperatively but is already existing for exact insertion (50). In addition, a statistical dependency showed that patients with a tumor disease developed more complications regardless of whether they were treated with a PSI or a CR.

When looking at the measurement distances, significant differences were found in one measurement point. The difference between the gonion (defined as point 6) experienced an average dimensional change of 1.52 mm (SD = 3.9) in the group of CR patients and differed significantly from the group PSI, which only showed average differences of -1.04 mm (SD = 4.9). Recent research identified additional dimensional changes in patients who were restored with CR (31). A changed distance between the mandibular and the intercondylar angle after using intraoperatively bended plates was documented (51). This goes in line with previous research indicating that better three-dimensional precision is achieved when using PSI (1, 16, 31, 44, 51). Dimensional changes due to rotation errors of the resection parts are avoided by prescribing exact positioning in the preoperative planning aiming at restoring the physiological position (51).

The division into four quartiles allowed a more specific examination of the extent to which the size of the defect depends on the diagnosis and predisposes to certain complications. In 24.0% of SCC patients, the resection extent was between 31 and 60 mm, thus in the second size quartile. Tumor size in oral squamous cell carcinoma is a risk factor for the development of systemic inflammation and postoperative complications (52). In size quartile two, where the patients with SCC are located, an increased incidence of dehiscence in the postoperative course was identified. In defect size quartile three, between 61 and 90 mm, most frequent complications were exposed plates in the healing process. The literature indicates that a low number of residual teeth is a significant prognostic factor for reconstruction plate loss (53). The results of this study are consistent with the literature as bone loss in the 61–90-mm range is associated with loss of dentition. The choice of reconstruction has no impact on plate exposure in the healing process (54). Other risk factors are rather intraoperative blood loss and the choice of reconstruction flap (55).

## Conclusion

For the reconstruction of mandibular defects, regardless of their etiology, the options available to the treating surgeon include CR and reconstruction using PSI. In each case, the advantages and disadvantages of the options must be weighed. Regardless of the method of fixation, the functional and esthetic outcomes of mandibular reconstruction have been significantly improved by the experience of the surgeon with microvascular grafts along with preoperative planning. In line with extant research, we highlighted that three-dimensional precision seems to be superior with a PSI. Interoperator variability can be reduced, and the training of younger surgeons involved in planning can be improved for better outcomes.

In future research, scholars might be interested in not only comparing the two methods used in this study but also validating the individual procedures. Thereby, the comparison of pre- and postoperative CT scans might be validated within the planning software (merging images). In a prospective study design, the required time for the individual virtual planning might be assessed along with relevant economic aspects, such as cost and time spent on patients receiving different surgical procedures.

## Data Availability Statement

The raw data supporting the conclusions of this article will be made available by the authors, without undue reservation.

## Ethics Statement

The studies involving human participants were reviewed and approved by local ethics committee at the University of Düsseldorf, Germany (Approval number 2018-250). Written informed consent for participation was not required for this study in accordance with the national legislation and the institutional requirements.

## Author Contributions

MR and HM designed and coordinated this study. HM and LA collected data. HM, LA, ER, and MW analyzed the data. HM and LA wrote this paper. MR, DS, and NK general supervised the research group. All authors contributed to the article and approved the submitted version.

## Conflict of Interest

The authors declare that the research was conducted in the absence of any commercial or financial relationships that could be construed as a potential conflict of interest.

## Publisher’s Note

All claims expressed in this article are solely those of the authors and do not necessarily represent those of their affiliated organizations, or those of the publisher, the editors and the reviewers. Any product that may be evaluated in this article, or claim that may be made by its manufacturer, is not guaranteed or endorsed by the publisher.

## References

[1] Essig H Rana M Kokemueller H von See C Ruecker M Tavassol F. Pre-Operative Planning for Mandibular Reconstruction - A Full Digital Planning Workflow Resulting in a Patient Specific Reconstruction. Head Neck Oncol (2011) 3(1):45. doi: 10.1186/1758-3284-3-45 21968330 PMC3195208

[2] Zhou L Shang H He L Bo B Liu G Liu Y. Accurate Reconstruction of Discontinuous Mandible Using a Reverse Engineering/Computer-Aided Design/Rapid Prototyping Technique: A Preliminary Clinical Study. J Oral Maxillofac Surg (2010) 68(9):2115–21. doi: 10.1016/j.joms.2009.09.033 20542365

[3] U V Mehrotra D Howlader D Singh PK Gupta S. Patient Specific Three-Dimensional Implant for Reconstruction of Complex Mandibular Defect. J Craniofac Surg (2019) 30(4):e308–11. doi: 10.1097/SCS.0000000000005228 31166275

[4] Chaine A Pitak-Arnnop P Hivelin M Dhanuthai K Bertrand J-C Bertolus C. Postoperative Complications of Fibular Free Flaps in Mandibular Reconstruction: An Analysis of 25 Consecutive Cases. Oral Surg Oral Med Oral Pathol Oral Radiol Endod (2009) 108(4):488–95. doi: 10.1016/j.tripleo.2009.05.043 19699114

[5] Eckardt A Swennen GRJ. Virtual Planning of Composite Mandibular Reconstruction With Free Fibula Bone Graft. J Craniofac Surg (2005) 16(6):1137–40. doi: 10.1097/01.scs.0000186306.32042.96 16327572

[6] Hallermann W Olsen S Bardyn T Taghizadeh F Banic A Iizuka T. A New Method for Computer-Aided Operation Planning for Extensive Mandibular Reconstruction. Plast Reconstr Surg (2006) 117(7):2431–7. doi: 10.1097/01.prs.0000219076.83890.e8 16772952

[7] Adolphs N Haberl E-J Liu W Keeve E Menneking H Hoffmeister B. Virtual Planning for Craniomaxillofacial Surgery – 7 Years of Experience. J Craniomaxillofac Surg (2014) 42(5):e289–95. doi: 10.1016/j.jcms.2013.10.008 24286863

[8] Wilde F Cornelius CP Schramm A. Computer-Assisted Mandibular Reconstruction Using a Patient-Specific Reconstruction Plate Fabricated With Computer-Aided Design and Manufacturing Techniques. Craniomaxillofac Trauma Reconstr (2014) 7(2):158–66. doi: 10.1055/s-0034-1371356 PMC4078148 25045420

[9] Rodby KA Turin S Jacobs RJ Cruz JF Hassid VJ Kolokythas A. Advances in Oncologic Head and Neck Reconstruction: Systematic Review and Future Considerations of Virtual Surgical Planning and Computer Aided Design/Computer Aided Modeling. J Plast Reconstr Aesthet Surg (2014) 67(9):1171–85. doi: 10.1016/j.bjps.2014.04.038 24880575

[10] Gil RS Roig AM Obispo CA Morla A Pagès CM Perez JL. Surgical Planning and Microvascular Reconstruction of the Mandible With a Fibular Flap Using Computer-Aided Design, Rapid Prototype Modelling, and Precontoured Titanium Reconstruction Plates: A Prospective Study. Br J Oral Maxillofac Surg (2015) 53(1):49–53. doi: 10.1016/j.bjoms.2014.09.015 25305795

[11] Antony AK Chen WF Kolokythas A Weimer KA Cohen MN. Use of Virtual Surgery and Stereolithography-Guided Osteotomy for Mandibular Reconstruction With the Free Fibula. Plast Reconstr Surg (2011) 128(5):1080–4. doi: 10.1097/PRS.0b013e31822b6723 22030490

[12] Powcharoen W Yang W-F Yan Li K Zhu W Su Y-X. Computer-Assisted Versus Conventional Freehand Mandibular Reconstruction With Fibula Free Flap: A Systematic Review and Meta-Analysis. Plast Reconstr Surg (2019) 144(6):1417–28. doi: 10.1097/PRS.0000000000006261 31764662

[13] Barr ML Haveles CS Rezzadeh KS Nolan IT Castro R Lee JC. Virtual Surgical Planning for Mandibular Reconstruction With the Fibula Free Flap: A Systematic Review and Meta-Analysis. Ann Plast Surg (2020) 84(1):117–22. doi: 10.1097/SAP.0000000000002006 31633539

[14] Serrano C van den Brink H Pineau J Prognon P Martelli N. Benefits of 3D Printing Applications in Jaw Reconstruction: A Systematic Review and Meta-Analysis. J Craniomaxillofac Surg (2019) 47(9):1387–97. doi: 10.1016/j.jcms.2019.06.008 31350034

[15] Nilsson J Hindocha N Thor A. Time Matters - Differences Between Computer-Assisted Surgery and Conventional Planning in Cranio-Maxillofacial Surgery: A Systematic Review and Meta-Analysis. J Craniomaxillofac Surg (2020) 48(2):132–40. doi: 10.1016/j.jcms.2019.11.024 31955991

[16] Rana M Chin S-J Muecke T Kesting M Groebe A Riecke B. Increasing the Accuracy of Mandibular Reconstruction With Free Fibula Flaps Using Functionalized Selective Laser-Melted Patient-Specific Implants: A Retrospective Multicenter Analysis. J Craniomaxillofac Surg (2017) 45(8):1212–9. doi: 10.1016/j.jcms.2017.04.003 28552201

[17] Urken ML Weinberg H Vickery C Buchbinder D Lawson W Biller HF. Oromandibular Reconstruction Using Microvascular Composite Free Flaps: Report of 71 Cases and a New Classification Scheme for Bony, Soft-Tissue, and Neurologic Defects. Arch Otolaryngol Head Neck Surg (1991) 117(7):733–44. doi: 10.1001/archotol.1991.01870190045010 1863438

[18] Hidalgo DA Pusic AL. Free-Flap Mandibular Reconstruction: A 10-Year Follow-Up Study. Plast Reconstr Surg (2002) 110(2):438–49. doi: 10.1097/00006534-200208000-00010 12142657

[19] Bak M Jacobson AS Buchbinder D Urken ML. Contemporary Reconstruction of the Mandible. Oral Oncol (2010) 46(2):71–6. doi: 10.1016/j.oraloncology.2009.11.006 20036611

[20] Rendenbach C Sellenschloh K Gerbig L Morlock MM Beck-Broichsitter B Smeets R. CAD-CAM Plates Versus Conventional Fixation Plates for Primary Mandibular Reconstruction: A Biomechanical In Vitro Analysis. J Craniomaxillofac Surg (2017) 45(11):1878–83. doi: 10.1016/j.jcms.2017.08.024 28943180

[21] Telschow T Wilde F Pietzka S Schramm A Mascha F. Unbreakable? – Frakturen Patientenspezifischer Implantate Nach Alloplastischer Unterkieferrekonstruktion. MKG-Chirurg (2019) 12(4):263–7. doi: 10.1007/s12285-019-00220-x

[22] Wilde F Hanken H Probst F Schramm A Heiland M Cornelius C-P. Multicenter Study on the Use of Patient-Specific CAD/CAM Reconstruction Plates for Mandibular Reconstruction. Int J Comput Assist Radiol Surg (2015) 10(12):2035–51. doi: 10.1007/s11548-015-1193-2 25843949

[23] Shan X-F Chen H-M Liang J Huang J-W Cai Z-G. Surgical Reconstruction of Maxillary and Mandibular Defects Using a Printed Titanium Mesh. J Oral Maxillofac Surg (2015) 73(7):1437.e1–9. doi: 10.1016/j.joms.2015.02.025 25971919

[24] Roser SM Ramachandra S Blair H Grist W Carlson GW Christensen AM. The Accuracy of Virtual Surgical Planning in Free Fibula Mandibular Reconstruction: Comparison of Planned and Final Results. J Oral Maxillofac Surg (2010) 68(11):2824–32. doi: 10.1016/j.joms.2010.06.177 20828910

[25] Hanasono MM Skoracki RJ. Computer-Assisted Design and Rapid Prototype Modeling in Microvascular Mandible Reconstruction. Laryngoscope (2013) 123(3):597–604. doi: 10.1002/lary.23717 23007556

[26] Hsu SS-P Gateno J Bell RB Hirsch DL Markiewicz MR Teichgraeber JF. Accuracy of a Computer-Aided Surgical Simulation Protocol for Orthognathic Surgery: A Prospective Multicenter Study. J Oral Maxillofac Surg (2013) 71(1):128–42. doi: 10.1016/j.joms.2012.03.027 PMC3443525 22695016

[27] Schendel SA Jacobson R Khalessi S. 3-Dimensional Facial Simulation in Orthognathic Surgery: Is it Accurate? J Oral Maxillofac Surg (2013) 71(8):1406–14. doi: 10.1016/j.joms.2013.02.010 23642546

[28] Stirling Craig E Yuhasz M Shah A Blumberg J Salomon J Lowlicht R. Simulated Surgery and Cutting Guides Enhance Spatial Positioning in Free Fibular Mandibular Reconstruction. Microsurgery (2015) 35(1):29–33. doi: 10.1002/micr.22229 24470389

[29] Weitz J Bauer F Hapfelmeier A Rohleder NH Wolff K-D Kesting MR. Accuracy of Mandibular Reconstruction by Three-Dimensional Guided Vascularised Fibular Free Flap After Segmental Mandibulectomy. Br J Oral Maxillofac Surg (2016) 54(5):506–10. doi: 10.1016/j.bjoms.2016.01.029 26898519

[30] Farfalli GL Albergo JI Ritacco LE Ayerza MA Milano FE Aponte-Tinao LA. What Is the Expected Learning Curve in Computer-Assisted Navigation for Bone Tumor Resection? Clin Orthop Relat Res (2017) 475(3):668–75. doi: 10.1007/s11999-016-4761-z PMC5289161 26913513

[31] Zeller AN Neuhaus MT Weissbach LVM Rana M Dhawan A Eckstein FM. Patient-Specific Mandibular Reconstruction Plates Increase Accuracy and Long-Term Stability in Immediate Alloplastic Reconstruction of Segmental Mandibular Defects. J Maxillofac Oral Surg (2020) 19(4):609–15. doi: 10.1007/s12663-019-01323-9 PMC7524954 33071511

[32] Tarsitano A Ciocca L Scotti R Marchetti C. Morphological Results of Customized Microvascular Mandibular Reconstruction: A Comparative Study. J Craniomaxillofac Surg (2016) 44(6):697–702. doi: 10.1016/j.jcms.2016.03.007 27107476

[33] Avraham T Franco P Brecht LE Ceradini DJ Saadeh PB Hirsch DL. Functional Outcomes of Virtually Planned Free Fibula Flap Reconstruction of the Mandible. Plast Reconstr Surg (2014) 134(4):628e–34e. doi: 10.1097/PRS.0000000000000513 25357057

[34] Moro A Cannas R Boniello R Gasparini G Pelo S. Techniques on Modeling the Vascularized Free Fibula Flap in Mandibular Reconstruction. J Craniofac Surg (2009) 20(5):1571–3. doi: 10.1097/SCS.0b013e3181b0db5c 19816298

[35] Nocini PF Saia G Bettini G Ragazzo M Blandamura S Chiarini L. Vascularized Fibula Flap Reconstruction of the Mandible in Bisphosphonate-Related Osteonecrosis. Eur J Surg Oncol (2009) 35(4):373–9. doi: 10.1016/j.ejso.2008.05.002 18562154

[36] Foster RD Anthony JP Sharma A Pogrel MA. Vascularized Bone Flaps Versus Nonvascularized Bone Grafts for Mandibular Reconstruction: An Outcome Analysis of Primary Bony Union and Endosseous Implant Success. Head Neck (1999) 21(1):66–71. doi: 10.1002/(SICI)1097-0347(199901)21:1<66::AID-HED9>3.0.CO;2-Z 9890353

[37] Pogrel M Podlesh S Anthony JP Alexander J. A Comparison of Vascularized and Nonvascularized Bone Grafts for Reconstruction of Mandibular Continuity Defects. J Oral Maxillofac Surg (1997) 55(11):1200–6. doi: 10.1016/S0278-2391(97)90165-8 9371107

[38] de Santis G Pinelli M Starnoni M. Extended and Unusual Indications in Jaw Reconstruction With the Fibula Flap: An Overview Based on Our 30-Year Experience. Ann Med Surg (2021) 62:37–42. doi: 10.1016/j.amsu.2020.12.049 PMC7806501 33489114

[39] Modabber A Ayoub N Möhlhenrich SC Goloborodko E Sönmez TT Ghassemi M. The Accuracy of Computer-Assisted Primary Mandibular Reconstruction With Vascularized Bone Flaps: Iliac Crest Bone Flap Versus Osteomyocutaneous Fibula Flap. Med Devices (Auckl) (2014) 7:211–7. doi: 10.2147/MDER.S62698 PMC4064953 24966700

[40] Ren W Gao L Li S Chen C Li F Wang Q. Virtual Planning and 3D Printing Modeling for Mandibular Reconstruction With Fibula Free Flap. Med Oral Patol Oral Cir Bucal (2018) 23(3):e359–66. doi: 10.4317/medoral.22295 PMC5945234 29680849

[41] Tiberio F Cacciotti I Frassanito P Nocca G Tamburrini G Arcovito A. Personalized Bone Reconstruction and Regeneration in the Treatment of Craniosynostosis. Appl Sci (2021) 11(6):2649. doi: 10.3390/app11062649

[42] Rustemeyer J Sari-Rieger A Melenberg A Busch A. Comparison of Intraoperative Time Measurements Between Osseous Reconstructions With Free Fibula Flaps Applying Computer-Aided Designed/Computer-Aided Manufactured and Conventional Techniques. Oral Maxillofac Surg (2015) 19(3):293–300. doi: 10.1007/s10006-015-0493-6 25861911

[43] Ritschl LM Mücke T Fichter A Güll FD Schmid C Duc JMP. Functional Outcome of CAD/CAM-Assisted Versus Conventional Microvascular, Fibular Free Flap Reconstruction of the Mandible: A Retrospective Study of 30 Cases. J Reconstr Microsurg (2017) 33(4):281–91. doi: 10.1055/s-0036-1597823 28099975

[44] Modabber A Legros C Rana M Gerressen M Riediger D Ghassemi A. Evaluation of Computer-Assisted Jaw Reconstruction With Free Vascularized Fibular Flap Compared to Conventional Surgery: A Clinical Pilot Study. Int J Med Robot (2012) 8(2):215–20. doi: 10.1002/rcs.456 22213406

[45] Vaira LA de Riu G Ligas E Deiana G Vacca G Massarelli O. Neck Dissection With Harmonic Instruments and Electrocautery: A Prospective Comparative Study. Oral Maxillofac Surg (2021) 25(1):75–9. doi: 10.1007/s10006-020-00897-w 32809161

[46] Cho RS Lopez J Musavi L Kachniarz B Macmillan A Badiei B. Computer-Assisted Design and Manufacturing Assists Less Experienced Surgeons in Achieving Equivalent Outcomes in Cranial Vault Reconstruction. J Craniofac Surg (2019) 30(7):2034–8. doi: 10.1097/SCS.0000000000005748 31306375

[47] Forman DE Maurer MS Boyd C Brindis R Salive ME Horne FM. Multimorbidity in Older Adults With Cardiovascular Disease. J Am Coll Cardiol (2018) 71(19):2149–61. doi: 10.1016/j.jacc.2018.03.022 PMC6028235 29747836

[48] Barnes PJ. Mechanisms of Development of Multimorbidity in the Elderly. Eur Respir J (2015) 45(3):790–806. doi: 10.1183/09031936.00229714 25614163

[49] Forman DE Rich MW Alexander KP Zieman S Maurer MS Najjar SS. Cardiac Care for Older Adults. Time for a New Paradigm. J Am Coll Cardiol (2011) 57(18):1801–10. doi: 10.1016/j.jacc.2011.02.014 PMC4942282 21527153

[50] Rana M Essig H Eckardt AM Tavassol F Ruecker M Schramm A. Advances and Innovations in Computer-Assisted Head and Neck Oncologic Surgery. J Craniofac Surg (2012) 23(1):272–8. doi: 10.1097/SCS.0b013e318241bac7 22337424

[51] Naros A Weise H Tilsen F Hoefert S Naros G Krimmel M. Three-Dimensional Accuracy of Mandibular Reconstruction by Patient-Specific Pre-Bent Reconstruction Plates Using an "in-House" 3D-Printer. J Craniomaxillofac Surg (2018) 46(9):1645–51. doi: 10.1016/j.jcms.2018.05.047 29983306

[52] Kågedal Å Rydberg Millrud C Häyry V Kumlien Georén S Lidegran M Munck-Wikland E. Oropharyngeal Squamous Cell Carcinoma Induces an Innate Systemic Inflammation, Affected by the Size of the Tumour and the Lymph Node Spread. Clin Otolaryngol (2018) 43(4):1117–21. doi: 10.1111/coa.13122 29679522

[53] Hirohata H Yanagawa T Takaoka S Yamagata K Sasaki K Shibuya Y. A Small Number of Residual Teeth After the Mandibular Resection of Oral Cancer Is Associated With Titanium Reconstruction Plate Exposure. Clin Exp Dent Res (2019) 5(5):469–75. doi: 10.1002/cre2.208 PMC6820575 31687179

[54] Nicholson RE Schuller DE Forrest LA Mountain RE Ali T Young D. Factors Involved in Long- and Short-Term Mandibular Plate Exposure. Arch Otolaryngol Head Neck Surg (1997) 123(2):217–22. doi: 10.1001/archotol.1997.01900020107016 9046293

[55] Fanzio PM Chang K-P Chen H-H Hsu H-H Gorantla V Solari MG. Plate Exposure After Anterolateral Thigh Free-Flap Reconstruction in Head and Neck Cancer Patients With Composite Mandibular Defects. Ann Surg Oncol (2015) 22(9):3055–60. doi: 10.1245/s10434-014-4322-1 25564168

